# Prognostic Implications of MALAT1 and BACH1 Expression and Their Correlation with CTCs and Mo-MDSCs in Triple Negative Breast Cancer and Surgical Management Options

**DOI:** 10.1155/2022/8096764

**Published:** 2022-01-19

**Authors:** Samah Said Elbasateeny, Mahmoud Abdou Yassin, Mohamed Mahmoud Mokhtar, Adel Mohamed Ismail, Huda Fathy Ebian, Samia Hussein, Sherin Attia Shazly, Mai Mohammed Abdelwabab

**Affiliations:** ^1^Pathology Department, Faculty of Medicine, Zagazig University, Egypt; ^2^General Surgery Department, Faculty of Medicine, Zagazig University, Egypt; ^3^Surgical Oncology Department, Ismailia Teaching Oncology Hospital, Egypt; ^4^Clinical Pathology Department, Faculty of Medicine, Zagazig University, Egypt; ^5^Medical Biochemistry and Molecular Biology Department, Faculty of Medicine, Zagazig University, Egypt; ^6^Obstetrics and Gynecology Department, Faculty of Medicine, Zagazig University, Egypt

## Abstract

**Background:**

Triple negative breast cancer (TNBC) is a biologically separate entity of breast cancer that cannot get benefits from targeted or endocrine therapy.

**Objective:**

To assess the expression of MALAT1 and BACH1, as well as monocyte-myeloid-derived suppressor cell (Mo-MDSC) levels and circulating tumor cell (CTC) count in TNBC to correlate these markers with the clinic-pathological criteria of TNCB patients and to evaluate their roles as predictive markers for selection of the patients that can be operated by oncoplastic conserving breast surgery.

**Methods:**

Eighty-eight TNBC were managed by modified doughnut breast oncoplastic surgery in early stages and by modified radical mastectomy for patients with late stages unsuitable for breast-conserving. All were examined for MALAT1 and BACH1 expression by immunohistochemistry and RT-PCR as well as Mo-MDSC levels and CTCs.

**Results:**

MALAT1 and BACH1 expressions are correlated with the larger size, lymph node, distance metastasis, and TNM staging (*p* < 0.05). CTCs ≥ 5 and high MO-MDSCs were significantly more in TNBC with MALAT1 and BACH1 overexpression. The survival study proved that DFS for patients with both positive expression of MALAT1 and BACH1 was shorter than that of one positive expression, and both negative expression *p* ≤ 0.001, CTCs ≥ 5, and high Mo-MDSCs are associated with poor outcomes. No significant difference between modified round block and modified radical mastectomy techniques as regards recurrence. However, all postoperative management outcomes were significantly better in patients operated by oncoplastic conserving breast surgery.

**Conclusion:**

BACH1 and MALAT1 expressions are significantly upregulated in TNBC. They are correlated with CTCs and Mo-MDCs, and all are associated with poor outcomes. Not all TNBC patients have a bad prognosis, patients negative for one of MALAT1 and BACH1 or both, have a slightly good prognosis, and so can be managed by breast oncoplastic conserving surgery.

## 1. Introduction

Breast cancer is a worldwide major health problem; in Egypt, it is considered the commonest cancer in female [1]. Triple negative breast cancer (TNBC) has been proved as a biologically separate entity, having special prognosis and behavior as it lacks the expression of estrogen receptor (ER), progesterone receptor (PR), and human epidermal growth factor receptor 2 (HER2). So, these patients cannot get benefits from targeted or endocrine therapy [2].

TNBC represents 9-21% of all breast cancer [3] and usually presented at younger age. Many researches were done to find out other treatment options for TNBC as it lacks effective targeted. BTB and CNC homology1 (BACH1) is a member of the Cap ‘n'Collar/basic region leucine zipper factor (CNC-bZip) family, and it is a heme-binding transcription factor [4]. BACH1 expression was found to be upregulated in TNBC patients' tumors [5, 6].

Metastasis-associated lung adenocarcinoma transcript 1 (MALAT1) is the most copious (~3000 copies/cell) nuclear-retained lncRNA. High expression of MALAT1 was suggested to be associated with a poor prognosis in breast cancer patients [7].

Tumors can escape immunosurveillance via monocyte-myeloid-derived suppressor cells (Mo-MDSCs) which inhibit excessive immune responses enhancing tumor progression [8]. They correlate with tumor progression, in different cancer forms [9], yet, their predictive and prognostic values in TNBC are currently unknown. High levels of Mo-MDSCs are usually associated with larger number of circulating tumor cells (CTCs) [10], which are cancer cells detected in the circulation. As CTCs are rare in early breast stages of cancer, concerns will be raised about their validity as a biomarker in TNBC [11].

Although modified radical mastectomy (MRM) is the gold standard operation for breast cancer, yet breast conserving surgery became the hope for managing breast carcinoma especially in young females. With early detection of the disease, many conserving oncoplastic breast surgery methods have been developed as round block technique; however, it has some drawbacks like scar later on, disturbing the shape of areola and breast asymmetry, and it is difficult to manage peripherally located tumor; modified round block technique (MRBT) is an oncoplastic procedure that can overcome these complications [12].

The aim of this study is to assess MALAT1 and BACH1 expression as well as Mo-MDSC levels and CTC count in TNBC trying to correlate these markers with the clinic-pathological criteria of TNCB patient and to evaluate their roles as predictive markers for selection of the patients that can be operated by breast oncoplastic conserving surgery as MRBT.

## 2. Materials and Methods

### 2.1. Patients and Clinical Information

This is a prospective cohort study, conducted in the Departments of General Surgery, Pathology, Clinical Pathology, Medical Biochemistry and Molecular Biology, Obstetrics and Gynecology, Zagazig University Hospitals, and in the Department of Surgical Oncology, Ismailia, teaching oncology hospitals, Egypt, during the period between January 2018 and March 2021.

Eighty-eight breast cancer cases were obtained from cases operated in General Surgery Department. Tissue samples were taken either by excisional biopsy or mastectomy. In the pathology department, the samples were diagnosed histopathologically and proved to be TNBC by routine immunohistochemical staining. Breast carcinoma cases were treated based on American Joint Committee on Cancer (AJCC) staging and indications [13]. In Clinical Oncology and Nuclear Medicine Department, the cases were followed up for a median of 17.5 months (range 6-40) every three months by regular visits, in which, clinical examination, pelvi-abdominal U/S, chest X-ray, and other investigations according to patients' complaints. For all patients, baseline clinico-pathological data were collected.

#### 2.1.1. Ethical Approval

The present study was approved by Zagazig University, institutional review board (ZU-IRB: 6423). We obtained informed consent from all cases who are included in the research about the use of their data and operation photos.

### 2.2. Surgical Procedure

**Figure 1:**
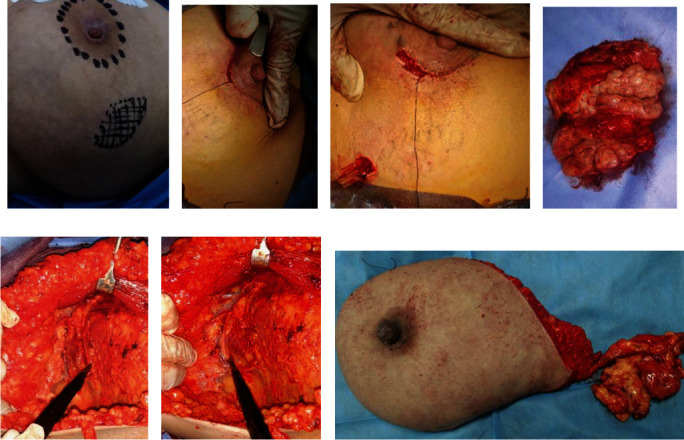
Surgical procedure: (a–d) modified round block technique; (e–g) modified radical mastectomy.

Patients with early TNBC, stage I (12) cases and stage IIA with no axillary lymph nodes (2) cases were operated by oncoplastic MRBT that is done by circum-areolar incision, subcutaneous dissection of the breast, and mobilization of its whole tissues that facilitate excision of the tumor with adequate surgical safe margin with no breast, nipple, or areola disturbance, while the patients with stage IIA with axillary lymphatic metastasis (6) patients and patients with stage IIIA (10) patients and stage IIIB (19) cases were operated by MRM; however, patients with stage IIIB with T4 (4) cases were managed by neoadjuvant downstaging then operated by MRM; however, patients with stage IV (10) cases were managed by palliative therapy as they were inoperable (Figure 1).

### 2.3. Immunohistochemical Staining and Evaluation

The sections (3–4 *μ*m) from tissue blocks were deparaffinized, followed by rehydration, then block the endogenous peroxidase activity. Antigen retrieval was carried out, then exposure to the primary antibody using streptavidin-biotin-peroxidase complex method for anti-MALAT1 antibody (dilution 1 : 500, Caiyou, Shanghai, China) and anti-BACH1 antibody (dilution 1 : 100, Santa Cruz Biotechnology, Europe), by applying diaminobenzidine (DAB) as the chromogen.

Staining intensity of cancer cells was judged to be MALAT-1 or BACH1 positive, if it was more intense than that of lymphocytes used as an internal control. Staining intensity was classified into three (weak: 0% ~10%, moderate: 11~49%, and strong: 50~100%). For statistical evaluation, weak staining was reports as negative, whereas moderate and strong staining were reported as positive. The negative controls for markers were done by primary antibody omission [14, 15].

### 2.4. RT-PCR

#### 2.4.1. RNA Extraction and Quantitative RT-PCR

Specimens of breast tissues were homogenized, followed by extraction of total cellular RNA from tissue homogenate by the use of easy-RED™ Total RNA extraction kit (iNtRON Biotechnology, Seongnam, Korea) following the manufacturer's instructions. Reverse transcription of one *μ*g of RNA was performed by the means of Maxime RT PreMix Kit (iNtRON Biotechnology, Seongnam, Korea) according to the manufacturer's protocol. qRT-PCR was performed by the add of 1 *μ*L of the cDNA, 100 pmol/*μ*L of each primer (1 *μ*L each) (Biolegio, Netherlands), 10 *μ*L of TOPreal™ qPCR 2x PreMIX (enzynomics), and 7 *μ*L RNAse free water. The real-time PCR was carried out by using Rotor-Gene Q 2 Plex (Qiagen, Hilden, Germany) as follows: 10 min of initial denaturation and polymerase activation at 95°C, after that 40 cycles of 95°C for 15 sec; annealing at 55°C for 15 sec; and finally elongation at 30°C for 45 sec.

We calculated the relative gene expressions for MALAT1 and BACH1 by using the 2 *ΔΔ*Ct method. We used GADPH as an internal control. Amplitudes of the change of the expression (fold change) observed in the tumors in relation to control adjacent tissues were analyzed by 2־*ΔΔ*Ct method [5, 16]. The primer sequence is listed in Table 1.

### 2.5. Flow Cytometry

EDTA peripheral blood samples (7.6 mL) were immediately transported for analysis at flow cytometry lab unit within 24 h after withdrawal from the patients. All flow cytometric analyses were performed on an FACS Caliber flow cytometer (BD Biosciences, San Diego, California, USA, FACScan flow) Cell Quest software for detection and identification of both Mo-MDSCs and CTCs. Modification of the method by Hristozova et al. [17] was used for CTC identification and counting. Analysis of Mo-MDSCs was done according to Bergenfelz et al. [18]; we used anti-human IgG as an isotype-matched negative control for each sample for both.

#### 2.5.1. Mo-MDSC Level

For flow cytometric detection of Mo-MDSCs, we incubated 100 *μ*L of blood sample for 20 min with 10 *μ*L of fluoroisothiocyanate (FITC) conjugated CD14, 10 *μ*L HLA-DR (peridinin-chlorophyll-protein complex) (Per-CP), and 10 *μ*L CD45 phycoerythrin (PE) (Becton Dickinson Biosciences, USA). After the lysis of RBCs and washing, the cells were resuspended in phosphate-buffered saline (PBS) then analyzed by flow cytometry. With each sample, forward and side scatter histograms were used to define the population of monocytes. After that, the expression of CD14 + ve was evaluated on monocyte population by gating on monocytes. Then, the expression of HLA-DR on CD14 + ve monocytes was detected, to define the CD14 + ve and HLADR +ve cells (Mo-MDSCs).

#### 2.5.2. CTC Count

Flow cytometric detection of CTCs was done by lysis of the erythrocytes in the remaining 7.5 mL blood, followed by incubation of the cell suspension for 20 min in the dark with cytokeratin FITC, CD326/EpCAM PE, and CD45 PE. We purchased monoclonal antibodies from Becton Dickinson Biosciences (San Jose, California, USA). The suspension is then washed with PBS, and then, the cells became ready for flow cytometric analysis. We identified and detected CTCs as CD45-ve, cytokeratin +ve, and EpCAM/CD326 + ve. Then, analysis of both the percentages and absolute counts of positive samples was performed. Our cases were alienated according to CTC count into two prognostic groups: the first one included patients with low CTC count (<5 cells/7.5 mL blood) while the second one included patients with high CT count (≥5 cells/7.5 mL blood).

### 2.6. Statistics

We calculated our sample size by using open Epi version 7. Data were analyzed by the use of IBM SPSS 23.0 for windows (SPSS Inc., Chicago, IL, USA) and NCSS 11 for windows (NCSS LCC., Kaysville, UT, USA). The quantitative data were expressed as mean ± standard deviation (SD), while qualitative data were expressed as percentage and frequency.

We used these tests: independent sample *t*-test, Mann–Whitney test for nonparametric data, *F* test (ANOVA) and Kruskal-Wallis for nonparametric data, chi-square for analysis of qualitative data, and Kaplan-Meier test for survival analysis. For probability (*p* value), when *p* value < 0.05, it was considered significant; if *p* value < 0.001, this was considered as highly significant, and when *p* value was >0.05, this was considered insignificant.

## 3. Results

### 3.1. Clinicopathological Features

The clinicopathologic characteristics of our TNBC 88 cases are summarized in Table 2.

### 3.2. BATCH1 and MALAT1 Immunohistochemical Expression and PCR Results

**Figure 2:**
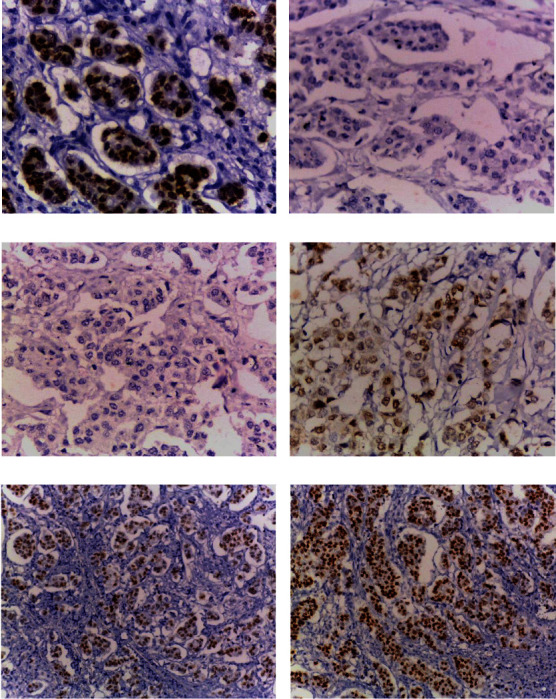
Representative samples of MALAT1 and BACH1 immunohistochemical expression in TNBC. (a) A case of positive MALAT1 immunoreactivity with strong nuclear expression (400x). (b) The previous case shows negative BACH1 expression. (c) A case of TNBC shows negative MALAT1 expression (400x). (d) The previous case shows positive BATCH1 immunoreactivity with moderate nuclear and cytoplasmic expression (400x). (e) A case of positive MALAT1 immunoreactivity with moderate expression (200x). (f) The previous case shows positive BATCH1 immunoreactivity with strong expression (200x).

MALAT-1 nuclear immunohistochemical expression and PCR positive cases were detected in 40.9% and 42%, respectively, of the studied TNBC cases, while BACH-1 immunohistochemical expression (mainly nuclear with occasional cytoplasmic reactivity) and PCR positive cases were detected in 55.7% and 56.8%, respectively, of the studied cases (Table 2, Figure 2).

The studied 88 TNBC specimens were classified into three groups according to the expression and positivity of MALAT1 and BACH1. Of these, 34.1% (30 out of 88) overexpressed (positive) both MALAT1 and BACH1. The results of correlation of marker expression with clinicopathological characteristic of the studied TNBC showed that MALAT1 and BACH1 expression (positivity) is correlated with size of the tumor, nodal metastasis, and TNM stage (*p* < 0.001; Table 3). Moreover, multiple comparisons were done for further investigation of the differences. The analysis showed that the expression in both negative vs. both positive groups (coexpression) of MALAT1 and BACH1 was correlated with the size of tumor, metastasis in LN, and TNM stage (*p* ≤ 0.000, 0.000, and 0.001, respectively; Table 4). Thus, we noticed that overexpression of MALAT1 and BACH1 was detected in TNBC with poor prognostic variables such as tumor size, nodal metastasis, and advanced tumor stage. This suggests that MALAT1 and BACH1 can have critical roles in carcinogenesis and progression of TNBC.

### 3.3. Association between CTCs and Other Parameters of the Study

**Figure 3:**
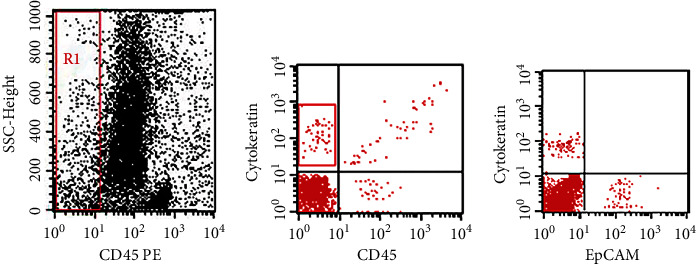
Flow cytometric detection of circulating tumor cells (CTCs). (a) CD45 and side scatter histogram was used to select the CD45 negative cells (R1). (b) The expressions of cytokeratin in CD45 negative. (c) CTCs were defined as EPCAM + cytokeratin + CD45-ve.

CTCs ≥ 5 showed significant association with disease stage, nodal metastasis, and distant metastasis (*p* ≤ 0.001, ≤ 0.001, and ≤ 0.001, respectively). CTCs ≥ 5 were significantly more frequent in TNBC cases with overexpression of MALAT1 and BACH1 (*p* < 0.001 and *p* < 0.001). CTCs ≥ 5 were significantly more frequent in TNBC patients who are relapsed or died (*p* < 0.001). No association was detected between CTCs and the grade of the tumors (Table 5, Figure 3).

### 3.4. Association between MO-MDSCs and Other Parameters of the Study

High MO-MDSC count was significantly more frequent with the overexpression of MALAT1, BACH1, more advanced stage, LN and distant metastasis, and the patients outcome *p* < 0.001, <0.001, <0.001, <0.001, < 0.001, and<0.001, respectively. High MO-MDSC count was significantly correlated with CTC ≥ 5 (*p* < 0.001). There was no statistically significant correlation between Mo-MDSCs and the grade of TNBC (Table 6).

### 3.5. Survival

**Figure 4:**
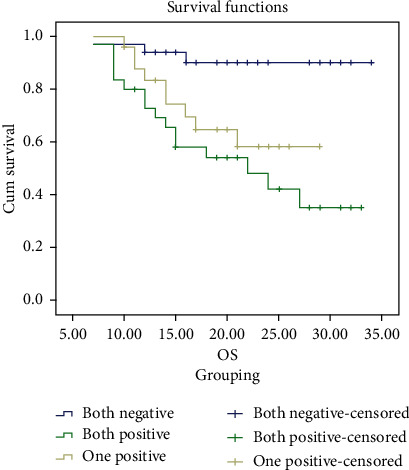
Kaplan-Meier OS analysis of studied cases according to MALAT-1 and BACH expression.

**Figure 5:**
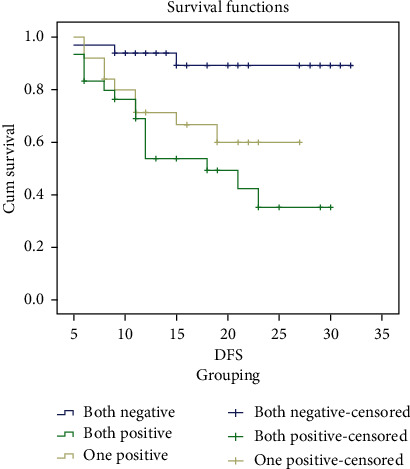
Kaplan-Meier DFS analysis of studied cases according to MALAT-1 and BACH expression.

The mean time of overall survival for patients with both positive expression was about 21 months, while mean period of OS for patients with one positive expression was about 23 months, with statistical significant difference log rank test (*p* ≤ 0.001). The disease-free survival mean time for patients with both positive expression was about 18 months, while mean period of DFS for patients with one positive expression was about 20 months, with statistically significant difference log rank test (*p* ≤ 0.001). CTCs ≥ 5 and high Mo-MDSCs are associated with poor outcome (Figures 4 and 5, Tables 7 and 8).

### 3.6. Surgical Analysis

Of the 88 TNBC studied cases, 14 patients underwent MRBT, while 60 patients were managed by MRM; four patients received neoadjuvant therapy before MRM; patients with stage IV breast carcinoma were inoperable; they managed by just palliative therapy (Tables 9 and 10).

Table 11 shows the postoperative outcomes of both techniques either MRBT or MRM. Postoperative recurrence developed in four (28%) patients operated by oncoplastic conserving surgery; however, it complicated 27 (45%) patients operated by MRM, with no significant difference. Postoperative wound infection was detected in only one case (7.5%) of the patients operated by MRBT, while it was detected in 11 (18.3%) patients of group operated by MRM with significant difference between both techniques. Patients operated by MRBT were satisfied by postoperative cosmetic results by 100%, while patients operated by MRM were satisfied by 71%, with significant difference between patients operated by both techniques. Postoperative drawback as seroma was detected in one patient (7.1%) and in 13 (21.7%) of patients managed by MRM with significant difference between both techniques. Postoperative pain was more observed in patients operated by MRM with highly significant difference.

## 4. Discussion

TNBC is considered a special category of breast cancer, which cannot get benefit from chemotherapy and other conventional therapeutic modalities [2].

MALAT1 is an early known lncRNA. Many researchers reported that MALAT1 plays a significant role in development and progression of numerous types of cancers [7, 19] and usually associated with poor prognostic factors especially in TNBC, signifying that MALAT1 could be considered an important prognostic factor and also target for therapy in these patients [20].

BACH1 is a transcription factor forming a heterodimer with the small Maf family proteins [4]; recent researches documented that BACH1 was overexpressed in TNBC [5, 6].

MO-MDSCs are a heterogeneous group of immunosuppressive cells which are usually enriched in various tumors and having a prognostic and predictive value for clinical outcomes. The role of MO-MDSCs in breast cancer patients remains relatively unexplored [10].

CTCs have a metastatic potential and can provide a noninvasive diagnostic blood test that provide distinctive information and could be used as an independent prognostic biomarker for metastatic breast cancer patients, but their predictive values in TNBC cases are not well fully investigated [21].

Therefore, the aim of this study was to evaluate the expression of MALAT1 and BACH1 as well as MO-MDSCs and CTCs in TNBC to evaluate the role of theses marker as a prognostic factors and potential targeted therapy of such TNBC.

In our research, we studied the pattern of MALAT1 and BATCH I expression as well as MO-MDSCs and CTCs in the studied 88 TNBC specimens. We found that MALATI and BACH1 were synchronously overexpressed in 34.1% of our studied cases. Moreover, correlation of marker expression with clinicopathological criteria detected that TNBC with overexpression of MALATI and BACH1 showed significantly larger size tumors, nodal metastasis, distant metastasis, and advanced stage. These findings were in concordance with previous related research performed with Ou et al. [14], who reported synchronous upregulation of MALAT1 and BATCH1 in 27% of studied TNBC and correlation of such expression with poor prognostic variables of studied TNBC cases.

Pervious researches reported that MALAT1 facilitate tumor cell proliferation, migration, metastasis, and invasion of blood-tumor-barrier [22]. Overexpression of MALAT1 is associated with larger sized tumors and advanced breast cancer stage [23] and poor outcome [20] particularly in TNBC [14]. Similar results of MALAT1 upregulation and promotion of tumor cell proliferation, migration, and invasiveness were detected in melanoma [24] and in endometrial carcinoma [23].

Earlier studies supported our finding of BACH1 upregulation in TNBC [5, 6]. Moreover, Han et al. [6] and Sato et al. [15] found that BACH1 favors cancer metastasis by promoting epithelial mesenchymal transition in cells of epithelial ovarian cancers and pancreatic cancers, respectively. Yun et al. [25] have concluded that BACH1 can promote bone metastasis in breast cancer through upregulation of metastatic genes, e.g., MMP1and CXCR4.

Based on analysis of our finding that is in concordance with many previous related and similar researches, we could infer that MALAT1 and BACH1 might participate in TNBC carcinogenesis and may have critical role in their progression and thus may be used in targeted therapy of such type of breast cancer.

We found that although CTCs ≥ 5 were associated with the advanced stages of the disease, some patients with early stages showed the presence of CTCs ≥ 5 suggesting the early spread of the disease which was in accordance with Elmorshidy et al. [26] who stated that the presence of CTCs in early stage disease implies that an advanced stage is not necessary for cancer cells to enter the circulation and spread.

We found that CTCs ≥ 5 was significantly associated with TNM staging as it was associated with advanced stage though it was present in early stages. Same results were obtained by Zhang et al. [27] who reported that CTCs are closely related to the TNM stage. Specifically, the higher the stage, the higher the positive detection rate of CTCs and the more possibility to suffer from distant metastasis. Recent study of Jin et al. [28] detected that more CTCs were found in patients with advanced TNM stage and in patients with invasive tumor or nodal metastasis.

In our study, CTCs ≥ 5 was significantly associated with the patient outcomes as frequency of CR was less and that of death was more with CTCs ≥ 5; these results are similar to the study of Zhang et al. [27] who concluded that CTCs can effectively predict TNBC recurrence and progression, which are helpful and have high values in judging and improving the prognosis. These results are supported also by Karhade et al. [29] who reported that CTCs predict shorter progression-free and overall survival in TNBC cases, and Gwark et al. [30] who found that patients with TNBC who failed to achieve PCR with ≥5 CTCs showed worse RFS and OS. Furthermore, CTCs could be used as an independent predictive and prognostic biomarker for MBC patients' outcomes [21] and can predict early disease recurrence and reduce the OS in patient's nonmetastatic breast cancer [26].

Our study revealed also that Mo-MDSC level was associated with disease stages and patient's outcome; this may be due to the role of these suppressor cells in inhibition of T-cell immune response against tumor cells. These results were supported by previous studies like Bergenfelz et al. [18] who found that Mo-MDSCs increased significantly during the course of disease progression and correlated to nodal metastasis, and they suggested that their monitoring may represent a simple novel biomarker for assessing disease progression. The patients with TNBC have increased levels of Mo-MDSCs that were higher in metastatic breast cancer [31]. Mo-MDSC was found to be related to metastatic or immunoregulatory switch associated with conversion to a more systemic disease, and high levels of systemic Mo-MDSCs represent patients with worse outcome and more aggressive disease [10].

High Mo-MDSC was associated with CTCs ≥ 5; this result was in accordance with that of Bergenfelz et al. [10] who stated that high Mo-MDSC levels were associated with more circulating tumor cells (CTCs), more metastatic sites, and with disease progression in cases of metastatic breast cancer.

In our study, we revealed that both of CTCs and Mo-MDSCs were associated with high expression of MALAT-1 and BACH-1, and all of them were associated with the progression of the disease and poor outcome.

As regards the management of our 88 TNBC cases, our surgical considerations stressed on operable patients and assessed the outcomes of types of operation. 14 patients operated by oncoplastic MRBT, and 60 patients were operated by MRM.

In our study, mean age was 37.6 ± 11.7. Zeeneldin et al. [32] published that the peak incidence of breast cancer is between 40 and 59 years.

For tumor size, it was between 0.5 and 8 with a mean of 3.99 ± 1.98. A systematic literature review of 474 articles from 55 studies evaluating 6011 patients was T1 (43.8%) and T2 (39.3%), and IDC were the most common tumor type [33]. According to another study in Rome, Italy, analyzing oncoplastic breast surgeries done over 20 years on 381 women, the IDC was the commonest; however, it was lower than that of our study as it was 54% [34].

The relapse peak after mastectomy as indicated by annual recurrence curves emerged in the first two years; however, recurrence after conservative breast surgery increased annually with the highest peak near five years as shown by most of the studies [35].

Our study showed recurrence in four patients operated by conservative breast surgery and 27 cases operated by MRM with no significant difference between both techniques. Patients with recurrence were referred to oncotherapy, and the four cases of round block techniques were reoperated by MRM. In study carried out by Abdl Rhaman et al. [36], none of the patients had any malignant recurrence.

Wound infection was detected in only one patient operated by MRBT and in 11 patients operated by MRM with significant better results in patients operated by MRBT. Patients with infected wound managed conservatively by systemic antibiotics. A study of Abdl Rhaman et al. [36] reported lower results of surgical site infection (10%) than reported by Vilar-Compte et al. [37] (18.9%) and higher than reported by Olsen et al. [38] (4.7%).

The present study showed seroma in only one patient operated by breast conserving surgery and in 13 patients managed by MRM with sig. difference; patients with seroma were managed by small puncture or aspiration, and there was no need for reoperation, while seroma developed in 9 patients (6.2%) in study by Refaat et al. [12].

Our study patients were satisfied by oncoplastic results (100%) more than patients operated by MRM (71.7%), while Ogawa [39] reported that fair and poor outcome occurred in 38.1% of patients in study carried by Refaat et al. in 2019 [12] found that 128/144 (88.9%) were satisfied. Pain was little observed significantly between patients operated by MRBT [40].

## 5. Conclusions

We can conclude that BACH1 and MALAT1 expression is significantly upregulated in TNBC. They are correlated with CTCs and Mo-MDCs, and all are associated with more progressive disease and poor outcome. Not all TNBC patients have bad prognosis, patients negative for one of MALAT1 and BACH1 or both markers, have slightly good prognosis, and so can be managed by breast oncoplastic conserving surgery. MRBT is one of oncoplastic breast surgery. It has several benefits as adequate tumor access and good aesthetic outcome.

## Figures and Tables

**Table 1:** The studied gene primer sequence.

	Forward	Reverse
BACH1	CACCGAAGGAGACAGTGAATCC	GCTGTTCTGGAGTAAGCTTGTGC
MALAT1	AAA GCA AGG TCT CCC CAC A A G	G G T C T G T G C T A G A T C A A A AGG
GADPH	A C C A C A G T C C A T G C C	T C C A C C C T G T T G C

**Table 2:** The clinicopathologic characteristics of the studied TNBC cases.

	All cases *N* = 88	
	Mean SD	
Age\years	37.6 ± 11.7	
OS time\months (range)	19.7 ± 7.5 (7–34)	
DFS\months (range)	16.6 ± 7.8 (5–32)	
Pathological type	*N*	%
IDC	78	88.6
Others	10	11.4
Grades		
I	12	13.6
II	40	45.5
III	36	40.9
Stage		
I	12	13.6
II a	13	14.8
II b	20	22.7
III a	10	11.4
III b	23	26.1
IV	10	11.4
Family history		
Positive	12	13.6
Negative	76	86.4
Menopause		
Pre-	64	72.7
Post	24	27.3
Perineural invasion		
Yes	41	46.6
LVI		
Yes	64	72.7
MALAT-1		
High (PCR)	37	42
Positive (IHC)	36	40.9
BACH-1		
High (PCR)	50	56.8
Positive (IHC)	49	55.7
Outcome		
CR	24	27.3
R	36	40.9
TNM stage		
Died	28	31.8
T		
Mean ± SD	3.99 ± 1.98 (0.5–8)	
*N*		
0-1	50	56.8
2-3	38	43.2
*M*		
0	78	88.6
1	10	11.4

**Table 3:** Relation between MALAT1 and BACH1 expression and basic characteristics of studied TNBC.

	Both -ve *N* = 33		Both +ve *N* = 30		One +ve *N* = 25			*p* value
Age\years	34.8 ± 10.7		38.6 ± 12.6		39.9 ± 10.3			
OS time\months (range)	21 (7-34)		10 (10-29)		15 (7-33)		6.63	0.04
DFS\months (range)	18 (5-32)		16 (6-27)		12 (5-30)		4.82	0.09
	*N*	%						
Pathological type								
IDC	31	93.9	25	83.3	22	88	1.77	0.44
Others	2	6.1	5	16.7	3	12		
Grades								
I	4	12.1	5	16.7	3	12	5.11	0.28
II	11	33.3	15	50	14	56		
III	19	57.6	10	33.3	8	32		
Stage								
I-II	29	87.9	7	23.3	9	36	29.4	<0.001
III–IV	4	12.1	23	76.7	16	64		
Family history								
Positive	5	15.2	6	20	1	4.0	3.07	0.22
Menopause								
Pre-	27	81.8	21	70	16	64	2.45	0.29
Post	6	18.2	9	30	9	36		
Perineural								
Yes	17	51.5	14	46.7	10	40	0.76	0.69
LVI								
Yes	22	66.7	23	76.7	19	76	0.98	0.61
MALAT-1								
High	0	0.0	30	100	7	28	67.3	<0.001
Positive	0	0.0	30	100	6	24	69.1	<0.001
BACH-1								
High	0	0.0	30	100	20	80	71.7	<0.001
Positive	0	0.0	30	100	19	76	69.5	<0.001
Outcome								
CR	14	42.4	4	13.3	6	24	15.8	0.003
R	16	48.5	10	33.3	10	40		
Died	3	9.1	16	53.3	9	36		
TNM stage								
T	1.8 (0.5–8)		4.75 (2.5-8)		4 (2.5-8)		21.4	<0.001
*N*								
0-1	29	87.9	10	33.3	11	44	21.4	<0.001
2-3	4	12.1	20	66.7	14	56		
*M*								
0	33	100	25	83.3	20	80	6.92	0.03
1	0	0.0	5	16.7	5	20		

NS: *p* value > 0.05 is not significant; S: *p* value < 0.05 is significant; HS: *p* value < 0.001 is high significant.

**Table 4:** Multiple comparisons.

	Both –ve vs. both +ve	Both –ve vs. one +ve	Both +ve vs. one +ve *p*
	*p*	*p*	
OS time\months	0.02	0.05	0.52
DFS\months	0.04	0.12	0.53
	*N*		
Grades	0.15	0.26	0.71
Stage			
I-II	<0.001	<0.001	0.31
III-IV			
Outcome			
CR	<0.001	0.04	0.38
R			
Died			
*N*			
0-1	<0.001	<0.001	0.42 NS
2-3			
*M*			
0	0.02	0.007	0.75
1			

NS: *p* value > 0.05 is not significant; S: *p* value < 0.05 is significant; HS: *p* value < 0.001 is high significant.

**Table 5:** Association between CTCs and basic characteristics among studied TNBC.

	<5 *N* = 51		>5 *N* = 37			
	*N*	%	*N*	%		
Grades						
I	6	11.8	6	16.2	1.92	0.38
II	21	41.2	19	51.4		
III	24	47.1	12	32.4		
Stage						
I	12	23.5	0	0.0	34.2	<0.001
II a	11	21.6	2	5.4		
II b	16	31.4	4	10.8		
III a	3	5.9	7	18.9		
III b	8	15.7	15	40.5		
IV	1	2.0	9	24.3		
MALAT-1						
High	9	17.6	28	75.7	29.6	<0.001
Low	42	82.4	9	24.3		
Positive	9	17.6	27	73.0	27.2	<0.001
Negative	42	82.4	10	27.0		
BACH-1						
High	16	31.4	34	91.9	Fisher	<0.001
Low	35	68.6	3	8.1		
Positive	15	29.4	34	91.9	Fisher	<0.001
	36	70.6	3	8.1		
Outcome						
CR	22	43.1	2	5.4	39.8	<0.001
R	26	51.0	10	27.0		
Died	3	5.9	25	67.6		
TNM stage						
T					MW	
Mean ± SD	5 (1.8–8)		3 (0.5–8)		3.86	<0.001
*N*						
0-1	40	78.4	10	27	23.1	<0.001
2-3	11	21.6	27	73		
*M*						
0	50	98.0	28	75.7	10.6	0.001
1	1	2.0	9	24.3		

**Table 6:** Association between monocytic MDSCs and basic characteristics among studied TNBC.

	Normal *N* = 51		High *N* = 37			
	*N*	%	*N*	%		
Grades						
I	6	11.8	6	16.2	5.11	0.08
II	19	37.3	21	56.8		
III	26	51.0	10	27.0		
Stage						
I	12	23.5	0	0.0	78.2	<0.001
II a	13	25.5	0	0.0		
II b	20	39.2	0	0.0		
III a	6	11.8	4	10.8		
III b	0	0.0	23	62.2		
IV	0	0.0	10	27.0		
MALAT-1						
High	14	27.5	23	62.2	10.6	0.001
Low	37	72.5	14	37.8		
Positive	15	29.4	21	56.8	6.62	0.01
Negative	36	70.6	16	43.2		
BACH-1						
High	19	37.3	31	83.8	18.9	<0.001
Low	32	62.7	6	16.2		
Positive	19	37.3	30	81.1	16.7	<0.001
Negative	32	62.7	7	18.9		
Outcome						
CR	23	45.1	1	2.7	29.8	<0.001
R	22	43.1	14	37.8		
Died	6	11.8	22	59.5		
TNM stage						
T					MW	
Mean ± SD	5 (1.8–8)		3 (0.5–8)		3.86	<0.001
*N*						
0-1	45	88.2	5	13.5	48.8	<0.001
2-3	6	11.8	32	86.5		
*M*						
0	51	100.0	27	73.0	15.6	<0.001
1	0	0.0	10	27.0		
CTCs						
< 5	40	78.4	11	29.7	20.9	<0.001
> 5	11	21.6	26	70.3		

**Table 7:** OS analysis of studied cases according to MALAT-1 and BACH expression.

	Estimated mean of overall survival\month	*p*
Both negative	31.8	0.001
Both positive	21.7	
One positive	22.96	

**Table 8:** DFS analysis of studied cases according to MALAT-1 and BACH expression.

	Estimated mean of overall survival\month	*p*
Both negative	29.7	0.001
Both positive	18.6	
One positive	20.6	

**Table 9:** Summary of surgical management of our cases.

Stage	No	L.N	Size	+ve MALAT1	+ve BACH1	Both -ve	Recurrence	Died	CR	Management
I	12	-ve	<2.5 cm	—	—	12	3	0	9	MRBT
II A	2	-ve	<2.5 cm	—	—	2	1	0	1	MRBT
II A	6	-ve	3-4.5	2	4	2	3	0	3	MRM
II A	5	+ve	1-3.5	—	—	5	0	0	5	MRM
II B	20	+ve or –ve	3-8	8	8	9	9	6	5	MRM
III A	10	+ve or –ve	3-7	7	10	—	7	2	1	MRM
III B	19	+ve or –ve	2.5-6	10	15	4	8	11	0	MRM
III B	4	+ve or –ve	Any size	3	2	1	3	1	0	Neoadjuvant then MRM
IV	10	+ve or –ve	2.5-7	6	9	—	2	8	0	Palliative therapy

**Table 10:** Marker expression in both studied patients.

Types Modified round block technique *N* = 14 *N* (%)		Modified radical mastectomy *N* = 60 *N* (%)	Total *N* = 74
+ve MALAT1	0 (0.0%)	27 (45%)	27 (36.4%)
+ve BACH1	0 (0.0%)	37 (61.7)	37 (50%)
-ve for both markers	14 (100%)	20 (33%)	34 (45.9%)

**Table 11:** Operative outcomes among both studied groups.

Types	Modified round block technique *N* = 14 *N* (%)	Modified radical mastectomy *N* = 60 *N* (%)		*p* value
Recurrence	4 (28.6%)	27 (45%)	*F*	0.26
Wound infection	1 (7.1%)	11 (18.3%)	Fisher	0.04
Patient satisfaction	14 (100%)	43 (71.7%)	Fisher	0.02
Seroma	1 (7.1%)	13 (21.7%)	Fisher	0.03
Pain			*X* ^2^	
Mild	10 (71.4%)	10 (16.7%)	17.4	<0.001
Moderate	3 (21.4%)	30 (50%)		
Severe	1 (7.1%)	20 (33.3%)		

## Data Availability

Data supporting the findings of the present research can be made available upon reasonable request from the corresponding author.

## Abbreviations

TNBC: Triple negative breast cancer
Mo-MDSCs: Monocyte-myeloid-derived suppressor cells
CTCs: Circulating tumor cells
MRBT: Modified round block technique
MRM: Modified radical mastectomy
RT-PCR: Reverse transcription polymerase chain reaction.
